# On Reflex Phenomena

**Published:** 1853-06

**Authors:** M. Cl. Bernard


From the Gazette Medicale.

*On Reflex Phenomena.* By M. Cl. Bernard.

Reflex Phenomena are of two kinds: the one having for object the accomplishment of the functions of organic life, the other those of animal life. M. Bernard has by his experiments endeavored to show that both of these phenomena are originally identical, and that the great sympathetic nerve plays a prominent part in their production. He first examined the reflex phenomena of organic life.

Two kinds of nerves are requisite for the production of these phenomena: the first transmits the impression to the nervous centres, the second to the viscera. With one order of these nervous filaments is always connected a ganglion of the great sympathetic; example: the ligual nerve transmits the impression of taste to the nervous centres, a special nerve then conveys a corresponding excitation to the submaxillary gland; on one of these nerves is situated a ganglion of the sympathetic, the submaxillary ganglion. —Another example: the optic nerve and the motores oculorum, the first transmitting the impression, the second the reflex excitation, are separated by the opthalmic ganglion. The pneumogastric nerve may be considered in the same light, in its relations with the liver, lungs, and spinal marrow; the last is here a conductor of reflex excitation, and the ganglion of the solar and cardiac plexuses play the same part as that which has been assigned in the preceding examples to those of the submaxilary and opthalmic regions.

Excite the lingual nerve, and the secretion of salva will be increased; cut the submaxilary ganglion, the nerve which connects it with the nervous centre, and the excitation of the lingual nerve will no longer produce this phenomenon. The experiments of Horbert Mayo prove that in the movements of the pupil, the results are identical as regards the optic nerve and the motores oculorum.

The pneumogastric nerve is the seat of analogous phenomena; as example: this nerve transmits to the nervous centre the impression made upon the lungs by atmospheric air, their habitual excitant; this impression, by means of the spinal marrow and sympathetic, immediately causes the production of sugar in the liver, which secretion, the result of reflex action, corresponds to the excitation of which the lungs are the seat, just as the secretion of saliva is the result of an irritation of the lingual nerve. Excite the lingual nerve and the secretion of saliva will be exaggerated; excite the lungs by means of chloroform or chlorine, and the secretion of sugar in the liver will be increased. From these considerations, M. Bernard is led to attribute the exaggerated production of sugar in the liver, caused by irritation of the pneumogastric nerve, to the reflex phenomena of organic life.

After having called the attention of the society to the important function which is performed by the great sympathetic in the production of visceral reflex phenomena, M. Bernard asks if the reflex phenomena of the limbs have not, even in this last point of view, a great analogy with the preceding; it seems at the first glance that such is not the case, for in the experiments of Marshal Hall, the ganglia do not appear to have any relation with the production of reflex movements. The following experiment, however, imagined by M. Bernard, seems to show that the intervertebral ganglia cannot be wounded without a corresponding cessation of reflex movement in the limbs. By a singular disposition the roots of the nerves of the anterior members in the frog, are seen, on dividing successively the muscles of the shoulder, to the outside of the medulary canal; the posterior provided with their proper ganglia. If these be examined attentively it will be observed that *all* the nervous filaments of the posterior root, do not traverse the ganglia, and that a certain number of them may be easily isolated by the point of a needle. From this anatomical arrangement it results that the ganglia may be destroyed and still a portion of the posterior root remain intact. Now if the section of the ganglia in the living frog be completed, reflex action ceases in the corresponding member; the animal no longer withdraws its foot on its being irritated; that sensibility, however, remains perfect, is proven by the fact that the animal manifests signs of pain by movements of the *whole* body. This experiment shows, then, that the intervertebral ganglia are necessary to the production of reflex movements in the limbs. Lead by analogy, M. Bernard concludes, in assimilating the intervertebral ganglia with those of the great sympathetic, that they are both necessary in the production of the two order of reflex phenomena admitted by him.

